# Fabrication and Manipulation of Ciliary Microrobots with Non-reciprocal Magnetic Actuation

**DOI:** 10.1038/srep30713

**Published:** 2016-07-29

**Authors:** Sangwon Kim, Seungmin Lee, Jeonghun Lee, Bradley J. Nelson, Li Zhang, Hongsoo Choi

**Affiliations:** 1 Department of Robotics Engineering, Daegu Gyeongbuk Institute of Science and Technology (DGIST), 711-873, Daegu, South Korea; 2 DGIST-ETH Microrobot Research Center, Daegu Gyeongbuk Institute of Science and Technology (DGIST), 711-873, Daegu, South Korea; 3 Institute of Robotics and Intelligent Systems, ETH Zurich, Zurich, CH-8092, Switzerland; 4 Department of Mechanical and Automation Engineering, Chinese University of Hong Kong, Shatin NT, Hong Kong SAR, China

## Abstract

Magnetically actuated ciliary microrobots were designed, fabricated, and manipulated to mimic cilia-based microorganisms such as paramecia. Full three-dimensional (3D) microrobot structures were fabricated using 3D laser lithography to form a polymer base structure. A nickel/titanium bilayer was sputtered onto the cilia part of the microrobot to ensure magnetic actuation and biocompatibility. The microrobots were manipulated by an electromagnetic coil system, which generated a stepping magnetic field to actuate the cilia with non-reciprocal motion. The cilia beating motion produced a net propulsive force, resulting in movement of the microrobot. The magnetic forces on individual cilia were calculated with various input parameters including magnetic field strength, cilium length, applied field angle, actual cilium angle, etc., and the translational velocity was measured experimentally. The position and orientation of the ciliary microrobots were precisely controlled, and targeted particle transportation was demonstrated experimentally.

Swimming microrobots can perform various biomedical operations, such as accurately targeted therapy, minimally invasive surgery, and precise cell or drug delivery by means of remote control in the fluidic environment of biological systems^1^,^2^,^3^. Many of these swimming microrobots have been studied with the intention of developing efficient motion control in fluidic environments, and for various biomedical applications^1^,^2^,^3^,^4^,^5^,^6^,^7^. Different types of microrobot features and accurate manipulation techniques are being continuously developed, and increasing the efficiency of locomotion for microrobots is an important design goal. Efficient swimming in a low Reynolds number environment requires innovative approaches for microrobots in terms of shape and actuating methodology on structure and mechanism design because of absence of inertia and presence of relatively high drag forces due to the small size of the microrobots^7^,^8^,^9^.

Biomimicry is one of the most effective approaches to achieving energy-effective structures and functionality on a small scale^8^,^9^,^11^,^12^,^13^,^14^,^15^,^16^,^17^,^18^,^19^,^20^,^21^,^22^,^23^,^24^,^25^,^26^,^27^. Microorganisms sometimes provide inspiration for efficient moving in a low Reynolds number fluidic environment. There have been several investigations of prokaryotic and eukaryotic flagellar and ciliary motion^11^,^12^,^13^,^14^,^15^,^16^,^17^,^18^,^19^,^20^,^21^,^22^,^23^,^24^,^25^,^26^,^27^. Prokaryotic flagella have a corkscrew-type motion with helical tails. To implement this motion, rotational magnetic fields were used^11^,^12^,^13^,^14^,^15^,^16^,^17^,^18^. The propulsion velocity of helical microrobots is determined by the drag forces, rotating frequency, intensity of magnetic fields, and geometrical parameters including the helix pitch. Eukaryotic flagella use a traveling-wave motion with a flexible tail. This motion is possible due to the external oscillating magnetic fields^19^,^20^,^21^,^22^,^23^,^24^,^25^. The velocity of the travelling wave motion is affected by drag forces and the bending stiffness of the tail. The travelling wave motion is possible due to the distributed actuation of the tail part; therefore it is more difficult to implement than helical propulsion^1^ or cilia that move with a power stroke motion^26^,^27^. The power stroke and recovery stroke motions have different actuating forces. These induce a net propulsive force, giving rise to movement. A key characteristic of these motions is the requirement of non-reciprocal motion within low Reynolds number fluids.

The design, fabrication and actuating methods for microrobots have been studied widely and realized with micro-/nano-technologies; magnetic manipulation is one of the popular methods for biomedical applications. The designs for locomotion of many microrobots have been motivated by microorganisms for efficient manipulation under an externally applied magnetic field^11^,^12^,^13^,^14^,^15^,^16^,^17^,^18^,^19^,^20^,^21^,^22^,^23^,^24^,^25^,^26^,^27^,^28^,^29^,^30^,^31^. Other mechanisms to manipulate micro-swimmers include biological and bio-hybrid approaches, which use a biological motor to achieve locomotion of microrobots^32^,^33^,^34^,^35^. However, such biological structures may have immune or toxicity issues, which may be unsuitable for *in vivo* applications. Also, the lifetime of microrobots based on a biological motor is relatively short and unstable in terms of material safety and reliability. Our main interests are magnetically actuated artificial microrobots with high propulsion, which will be efficient in many potential applications. An artificial ciliary motion is an actuation mechanism that mimics non-reciprocal ciliary beating motions. It has not been reported before because it is difficult to fabricate a ciliary structure with a high aspect ratio. Thus, we propose an artificial ciliary microrobot, to be actuated by non-reciprocal actuation for net positive propulsion that mimics the cilium beating of paramecium.

In a low Reynolds number environment, time-symmetric movement cannot create a net propulsive force, as can be explained with Purcell’s scallop theorem^8^. Thus, non-reciprocal motion of microrobots is required for effective locomotion within Newtonian liquids. Non-reciprocal motion can be achieved in one of two ways: a non-symmetric structure of the microrobot or non-symmetric actuation. For the first case, even though the actuating force is applied in a consistent and time-symmetric manner, a microrobot can move in a non-reciprocal way if the shape of the microrobots has chirality; i.e., is helical^11^,^12^,^13^,^14^,^15^,^16^,^17^,^18^ or has some other non-symmetrical shape^36^,^37^. Artificial helical micro-swimmers using a corkscrew motion have been developed and their motions are realized by magnetic actuation. Magnetic locomotion was demonstrated by rotating a magnetic field, which generated a magnetic torque along the rotational long axis of the helical microrobots for a corkscrew thrust. Secondly, non-reciprocal motion can also be generated from non-reciprocal actuation. Micro-organisms using a traveling wave motion and a ciliary stroke motion may not have chirality in their shape, but they can generate a propulsive force by a non-symmetric actuating motion^8^. If a non-symmetric actuation magnetic field can be applied, non-reciprocal motion is possible even with a symmetric structure. Microrobots using non-reciprocal motion due to non-reciprocal actuation have not been developed to our knowledge, probably because of the difficulty of implementing non-symmetrical actuation and fabricating the complicated ciliate structures.

In this paper, microrobots with cilia were designed and fabricated. The ciliary microrobots were developed to mimic cilia-based microorganisms, such as paramecium^26^,^27^. The structures were fabricated by 3D laser lithography^10^,^15^,^17^,^18^,^38^,^39^, and nickel and titanium thin layers were partially deposited only on the cilia parts, by sputtering the microrobot with a mask structure, for magnetic manipulation and biocompatibility, respectively. The position and orientation control was achieved using a magnetic manipulator with non-reciprocal actuation and stepping magnetic fields (on-off fields with designated angle). The actuation force was calculated and the translational velocity was measured. The fabricated ciliary microrobot was used to demonstrate targeted particle transportation using a micro-particle. The microrobots have a highly efficient propulsion mechanism in a low Reynolds number fluid environment.

## Results

### Design and fabrication of ciliary microrobots

**Figure 1:**
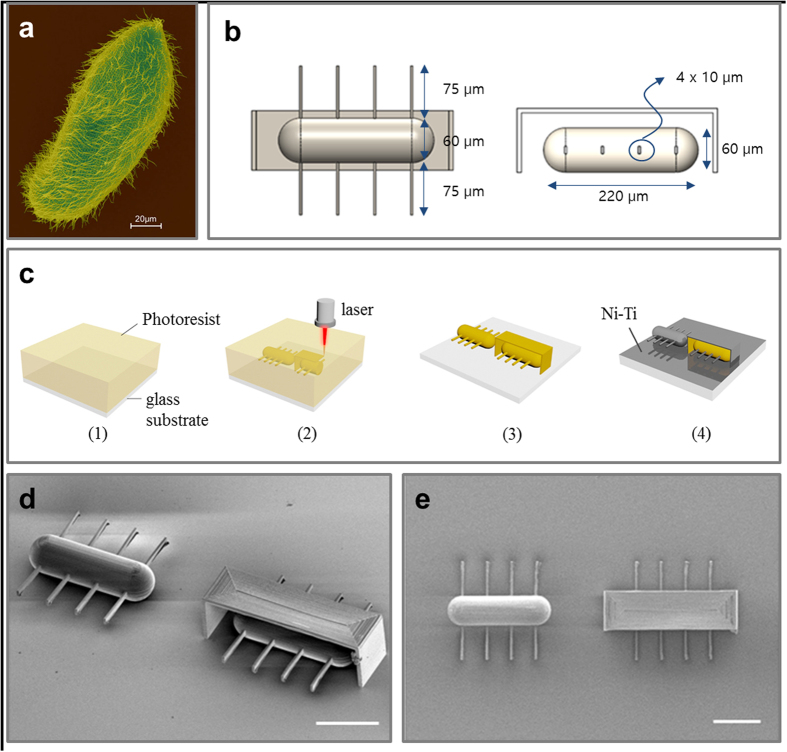
Design and fabrication of microrobots. (**a**) SEM image of microorganism, Paramecium, using ciliary stroke motion^27^. (**b**) Design layouts for artificial ciliary microrobots. (**c**) Overall fabrication process for the ciliary microrobot. SEM images of fabricated ciliary microrobots with and without a mask structure in trimetric view (**d**) and top view (**e**). (The scale bar is 100 μm) Reproduced with permission^27^, Copyright 2011, Springer.

**Figure 2:**
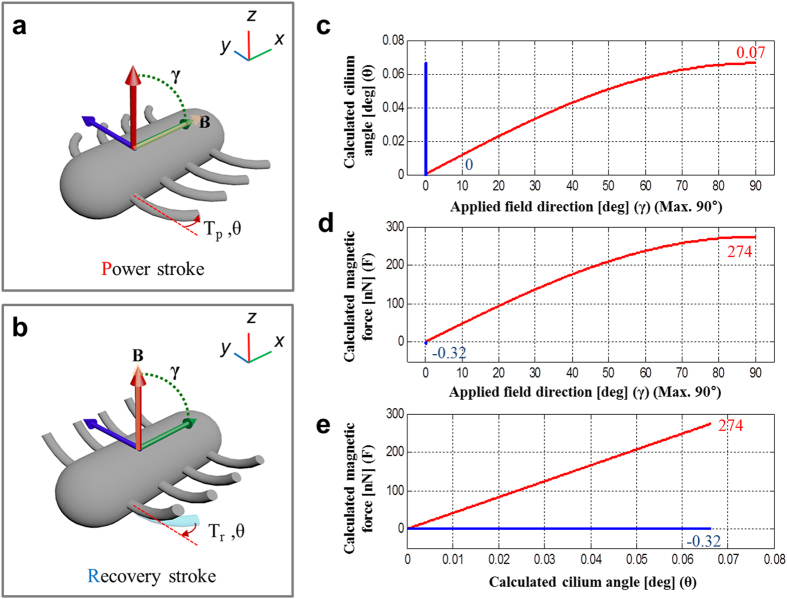
Schematics of magnetic actuation for the ciliary microrobot and the calculated actuating magnetic force. The external magnetic field (B) is shown along with the stepping angle in the non-axial symmetric range (0°–90° in the *x*-*y* plane). Magnetic torque is considered only in the *x*-*y* plane to generate a force along the *x*-direction. (**a**) Power stroke motion with T_p_, and (**b**) recovery stroke with T_r_, which generate two different magnetic actuation forces. (**c**) Applied field direction vs. calculated cilium angle, (**d**) applied field direction vs. calculated magnetic force, and (**e**) cilium angle vs. calculated magnetic force (the red line is the power stroke and the blue line is the recovery stroke).

**Figure 3:**
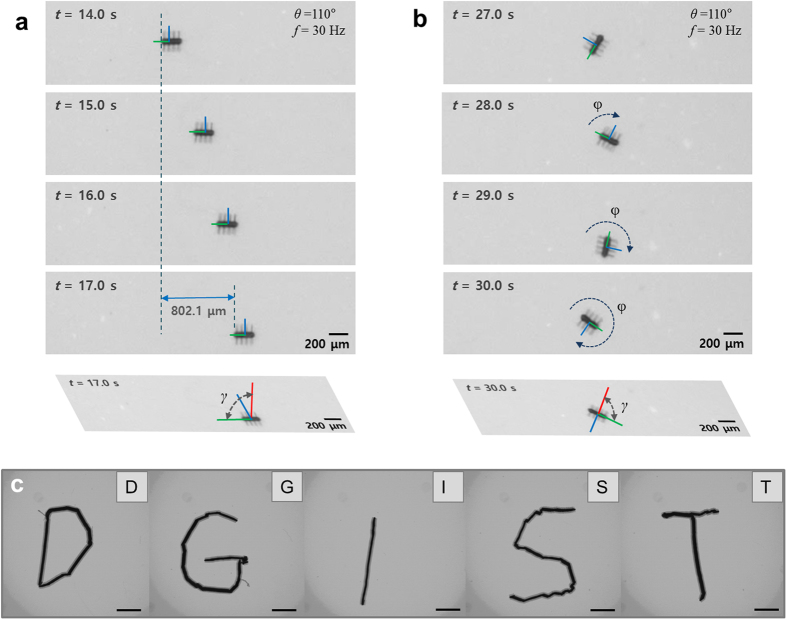
Time-lapse images during the manipulation of the cilia microrobot. The position and orientation were controlled precisely by applying stepping field (γ) to change the steering axis (φ). (**a**) Translational motion. (**b**) Rotational motion (see Supplementary Movie 1). (**c**) The microrobot was driven to write each letter of “DGIST”. The image was acquired by superposing each image from the video for each letter. The applied field intensity was 8 mT and we used a 35 Hz stepping field with a stepping angle of 110°. The scale bar is 1,000 μm (see Supplementary Movie 2).

**Figure 4:**
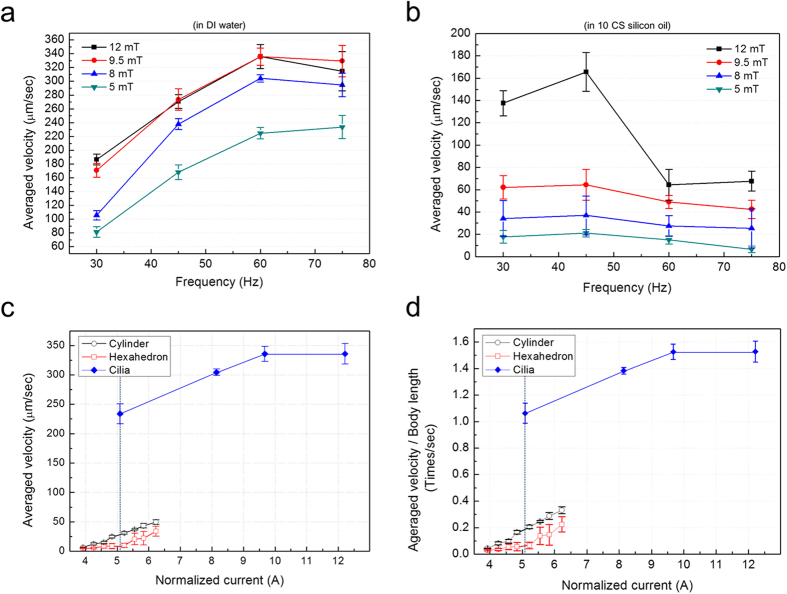
Velocities of various microrobots. (**a**) Averaged velocities (*n* = 3) of the ciliary microrobots with 110° stepping angle in DI water by the magnetic field control system. (**b**) Averaged velocities (*n* = 3) of the ciliary microrobots in 10 CS silicon oil, with a stepping angle of 110° due to the magnetic field control system. (**c**) The velocities of the ciliary microrobots compared to the velocities of the cylindrical and hexahedral microrobots in DI water. (**d**) The velocity of the microrobots in DI water, normalized to the body length.

Ciliary microrobots were designed and fabricated to mimic the paramecium, as shown in Fig. 1(a). The design of the ciliary microrobots was developed with a three-dimensional (3D) CAD tool (Solidworks, Dassault Systèmes SolidWorks Corp., USA). Design layouts of ciliary microrobots are shown in Fig. 1(b). The total body length of the microrobot is 220 μm and the body diameter is 60 μm. Four cilia are attached on each side of ellipsoidal body and the cilium length is 75 μm, with a cross-sectional area of 4 × 10 μm^2^. Additional mask structures were also designed to prevent the deposition of metal layers on the body. The designed cilia microrobots were fabricated by MEMS fabrication techniques, which include 3D laser lithography and metal sputtering. The overall fabrication procedure is shown in Fig. 1(c). 3D laser lithography system (Photonic professional, Nanoscribe GmbH, Germany) was used to build the structures using a polymer-based photoresist (IP-dip, Nanoscribe GmbH, Germany). The IP-dip photoresist was applied on the glass substrate (Fig. 1(c-1)) and a laser spot selectively polymerizes the photoresist (Fig. 1(c-2)) as the 3D CAD design. The exposed part of the photoresist remains after the development process (Fig. 1(c-3)). The 200 nm nickel and 20 nm titanium layers were partially deposited only on the cilia parts using a sputtering system (SORONA Co. Ltd., Korea) as shown in Fig. 1(c-4). Figure 1(d,e) show SEM images of the fabricated cilia microrobots with and without the mask structure. Metal layers are partially deposited only on the cilia parts due to the mask structures. (See ‘Methods’ section for the detailed fabrication techniques and recipes).

### Magnetic manipulation of microrobots

Manipulation of the microrobots was conducted with an electromagnetic actuator with eight coils (Minimag, Aeon Scientific GmbH, Switzerland)^40^. This hemispherical-configured electromagnetic system can apply stepping fields for translation of the microrobot, and can tilt the reference axis of the stepping fields to change the orientation of the microrobot. Figure 2(a,b) shows the principle of ciliary microrobot actuation for the generation of net displacement by the non-axial symmetric beating motion of the cilia. The cilium moves in 3D space according to the external stepping fields, beating on backwards and upwards trajectories. We calculated the x-y planar force components of the force generated by a cilium, as this is the net force contributing to translation. Therefore, the cilium angle (θ), and magnetic force (F) are projected components on the x-y plane. The relationships between the applied magnetic field direction (γ), θ and F can be calculated using a simple cantilever deflection method and the results calculated are shown in Fig. 2(c–e). The detailed calculation procedure is explained in the “Method” section. The maximum force during the power stroke was 274.40 nN at cilium angle of θ = 0.07°, and the maximum force during the recovery stroke was 0.32 nN at cilium angle of θ = 0° in the opposite direction to the power stroke. During the power stroke and recovery stroke stages, the angles between θ and γ were almost 90° and 0°, respectively. Thus, a magnetic torque difference occurs, as expected, due to Equation (1). This force difference causes non-reciprocal actuation, which is similar to the actual beating behavior of a cilium^27^.

Figure 3(a,b) shows time lapse images of the position and orientation control for a ciliary microrobot (Supplementary Movie 1). The stepping fields were applied at 0–110° to change the polar angle for translation, and the azimuthal angle (φ) was changed for rotation. The stepping angle was tuned for the best controllability at 110° in an actual experiment. However, this does not mean the cilia are actually moving in this range of angle. Because of the bending stiffness of the cilia, the cilia actually move much less than the applied oscillating angle, as shown by the calculated results using Equation (3), shown in Fig. 2(d). Figure 3(c) shows the manipulation of a microrobot, which drives along each letter of “DGIST”; the five videos are merged. This result shows the precise position and orientation control obtained using the microrobots we have developed (Supplementary Movie 2).

**Figure 5:**
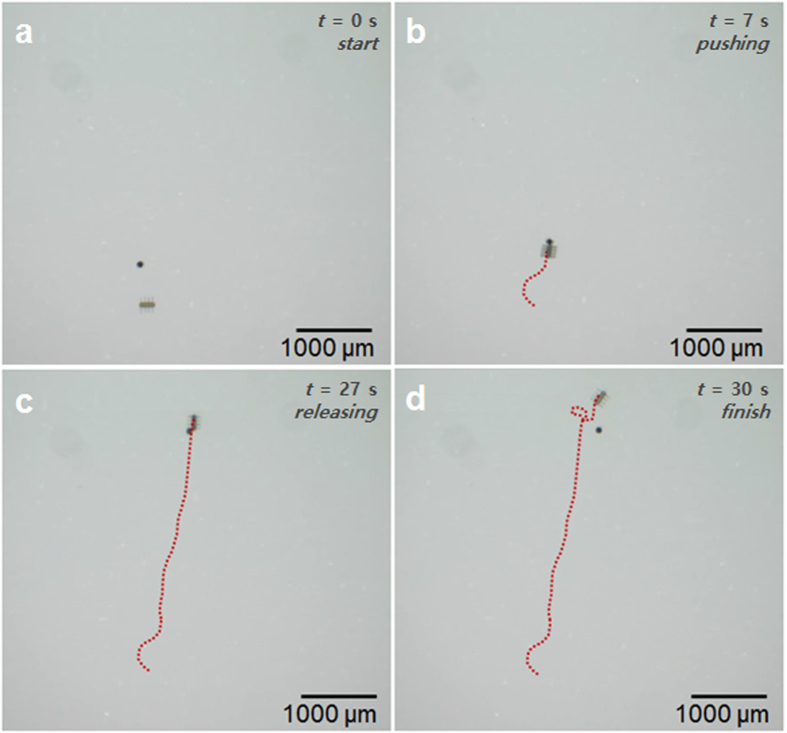
Time lapse images of the targeted micro-particle transportation with the ciliary microrobot. (**a**) The microrobot started and changed orientation to catch the micro-particle on the front side, and (**b**) the microrobot pushed the micro-particle with a translational motion. (**c**) The microrobot rotated back and forth to release the micro-particle, and finally (**d**) the microrobot moved away from the micro-particle. The diameter of the microsphere was 80 μm. The diameter of the micro-particle was 80 μm. The applied field intensity was 12 mT and we used a 35 Hz stepping field with a stepping angle of 110° (see Supplementary Movie 3).

The translational velocity of ciliary microrobots can be changed by controlling the frequency and the external magnetic field intensity. Figure 4(a) shows the measured velocities of the microrobots under different magnetic frequencies and magnetic field intensities in DI water. The maximum average velocity of the microrobots in DI water was 340 μms^−1^ (1.55 body lengths per second) under both 9.5 mT and 12 mT field intensities at 60 Hz; this is the saturation velocity. Figure 4(b) shows the measured velocities of the microrobots at various frequencies and applied magnetic fields in 10 CS silicon oil (KF-96 10 CS Silicon Fluid, Shin-Etsu Chemical Co., Ltd., Japan). The maximum average velocity was 165 μms^−1^ (0.75 body lengths per second) with a magnetic field of 12 mT, and at 45 Hz. Both the maximum velocity and the step-out frequency were smaller in silicon oil than in DI water due to the higher viscosity. Figure 4(c) shows a comparison with the microrobot velocities reported in ref. 4. The magnetic field gradient was used to obtain translational motion of cylindrical and hexahedral microrobots. The velocities are shown as a function of the normalized applied current in the eight electromagnetic coils of the magnetic control system. Therefore, these results could be interpreted as indicating the propulsion efficiency of each microrobot. The velocity of the ciliary microrobot was 233 μms^−1^, which is about 8.6 times and 25.8 times faster than the cylindrical and hexahedral microrobots, respectively, when the normalized current was 5.09A, as indicated by the blue dashed line in Fig. 4(c). Therefore, the translational motion of the ciliary microrobots is much more efficient than that of the previously reported cylindrical and hexahedral microrobots, even though the three microrobots have a similar characteristic body length (150 μm for cylindrical and hexahedral types of microrobots in the previous work [10], and 220 μm for ciliary microrobots reported in this paper). Figure 4(d) shows the translational velocities normalised to the body length of each microrobot. The ciliary microrobots exhibited more efficient translational motion than previously reported for cylindrical or hexahedral microrobots. When the ciliary microrobot moved in a circular path, the starting and ending points deviated by about 200 μm, which is smaller than the body length. In Fig. 5, we show the targeted particle transportation by the ciliary microrobot, in the case of a micro-particle with a diameter of 80 μm. The micro-particle was translated as it was pulled by the microrobot, and it was then released by the rotational motion of the microrobot. The applied field intensity was 12 mT and the stepping field frequency was 35 Hz, with a stepping angle of 110° (See Supplementary Movie 3).

## Discussion

The aim of this research was to implement ciliary microrobots, which swim in viscous fluid with non-reciprocal actuation, mimicking the movement of cilia. Other effective mechanisms to induce motion in microrobots, such as corkscrew motions in the case of chiral structures, and travelling wave motions in tails attached to the microrobots, have already been implemented. However, cilia-inspired microrobots are rarely reported, which might be due to the difficulty of fabricating the paramecium-like structures and achieving the non-reciprocal magnetic actuation. In this paper, ciliary microrobots were successfully fabricated using 3D laser lithography. They were manipulated by a stepping magnetic field using a magnetic manipulator. Non-symmetric switching of the magnetic fields in the range of a 110° angle in the polar direction was used to move the cilia with a non-axial symmetric actuation force, as the cilia tend to align with the applied magnetic field direction during the stroke motion. The steering axis (azimuthal angle) can also be changed to control the orientation of the microrobots. The reference axis of the oscillating stepping field can be changed via the azimuthal angle to change the orientation of the ciliary microrobot. Therefore, the position and orientation can be independently and precisely controlled at any time.

To enable magnetic control of the microrobots with cilia beating, a metal sputtering system was used to selectively deposit nickel and titanium layers onto the cilia parts by using mask structures. Sputtering is a conformal coating, guided by a shadow structure that was very close in shape to the ciliary microrobot. Because of the shadow structure, only a small amounts of metal were deposited onto the body of the microrobot. The magnetic field gradient was applied to pull the microrobots, but the robots did not move at all. This means that the total amount of magnetic material was not sufficient to generate a magnetic force that was able to translate the whole body by magnetic pulling using a magnetic field gradient at 1,000 mT/m. This shows that there was a very small amount of magnetic material on the body of the microrobot.

We confirmed the biocompatibility of the materials used in the proposed microrobots in previous cell culture experiments, in which we used various microrobots made from the same materials and on the same scale range (less than 300 μm)^10^. Therefore, we are convinced that the materials, shape and size of our microrobots are biocompatible. However, we may need to do a long term biocompatibility or toxicity test in an *in vivo*/*in vitro* environment. The issue of toxicity is important and should be studied in the future to determine whether our microbots are suitable for *in vivo* biomedical applications.

In this research, we developed cilia-inspired microrobots. The position and orientation of the fabricated microrobots were manipulated magnetically in DI water and 10 cs of silicon oil. The external stepping magnetic field generates an actuating force, which results in different stroke patterns of the cilia during the power stroke and recovery stroke. The translational velocities were evaluated by changing the frequency and applied magnetic field intensity. Locomotion of ciliary microrobots was first demonstrated, and this propulsion mechanism represents a high-efficiency swimming method for microrobots.

## Methods

### Fabrication of microrobots

Microrobots were fabricated using MEMS fabrication technology. Glass wafers of 30 mm diameter were cleaned as a starting substrate. The IP-dip photoresist (negative type) drop was at the center of the glass and the substrate was placed in a 3D laser lithography system for the laser writing process (Fig. 1(c-1)). The designed structures were loaded and the laser writing was performed. The wave length of the laser was 780 nm, and a two-photon polymerization method was used. The laser spot was scanned to polymerize the photoresist by precisely moving the piezoelectric stage (Fig. 1(c-2)). The optimized laser power and average scan speed were 8.0 mW and 70 μms^−1^, respectively. After laser writing, the substrate was developed by SU-8 developer for 10 min and the sample was rinsed with IPA (Fig. 1(c-3)). After drying the microrobot structures, 200 nm nickel and 20 nm titanium were deposited on microrobots sequentially with the sputtering system (Fig. 1(c-4)).

### Actuation force evaluation

The cilia beating cycle generates a net translational force due to the difference in the magnetic actuation force between the power stroke and the recovery stroke. The cilium angle (θ), and magnetic force (F) were projected onto the x-y plane to calculate the in-plane translational force of the ciliary microrobot. When an external magnetic field is applied along the long axis of the microrobot body at angle γ, which gradually increases from 0 to 90°, the cilia are bent, as shown in Fig. 2(a) with cilium angle θ. The applied field direction was 0° in the recovery stroke stage, and a force was generated due to the difference between the maximum cilium angle and 0°. To calculate the actuation force, we assumed the cilium angle was 0° after one beating cycle. The magnetic torque (**T**_m_) of a cilium with an external magnetic field is described by the following equation^41^:


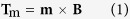


where **m** is the magnetic moment of a cilium, and **B** is the magnetic field intensity. This has a maximum value when the field is applied orthogonally to the magnetic moment. The equation for the magnetic force on a cilium along the longitudinal axis of the cilium body can be simplified during the backward (power) and forward (recovery) strokes to the following equation if the cilium length is L, the applied field angle is γ, and actual cilium angle is θ^42^:


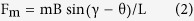


Only the planar (*x*-*y* plane) force components are counted in this calculation, and this only affects the body translation along the *x*-direction. The cilium angle is evaluated by a simple cantilever deflection model, as follows:


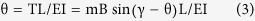


where *T* is the magnetic torque of the planar component of the ciliary microrobot, *E* is Young’s modulus of the cilium, and *I* is the moment of inertia. The relationships among the applied magnetic field direction (γ), cilium angle (θ), and magnetic force (F) were calculated using geometrical parameters and the material properties are listed in Table 1. Figure 2(c–e) shows the relationships among the applied magnetic field direction (γ), cilium angle (θ), and magnetic force (F). Figure S1 also shows in detail the temporal working mechanisms in one beating cycle.

## Additional Information

**How to cite this article**: Kim, S. *et al*. Fabrication and Manipulation of Ciliary Microrobots with Non-reciprocal Magnetic Actuation. *Sci. Rep*. **6**, 30713; doi: 10.1038/srep30713 (2016).

## Supplementary Material

Supplementary Information

Supplementary Movie S1

Supplementary Movie S2

Supplementary Movie S3

## Figures and Tables

**Table 1:** Parameters for calculating the actuation force.

Parameters	Value	Parameters	Value
Young’s modulus (E)	4.0 GPa^43^,^44^	Magnetization (M)	686,000 A/m
Cilium width (b)	4 μm	Cilium volume (V)	b*h*L
Cilium height (h)	10 μm	Moment of inertia (I)	bh^3^/12
Cilium length (L)	75 μm	Magnetic field intensity (B)	10 mT
		Magnetic moment (**m**)	V* M
