# Supplementary material for: Fabrication and Manipulation of Ciliary Microrobots with Non-reciprocal Magnetic Actuation
Source: Sci Rep. 2016 Jul 29;6:30713. doi: 10.1038/srep30713 (PMC4965827; doi:10.1038/srep30713)
Supplement: Supplementary Information [file srep30713-s1.pdf]

Supplementary Information

Manuscript title: Fabrication and Manipulation of Ciliary Microrobots with Non-reciprocal Magnetic Actuation

Authors: Sangwon Kim, Seungmin Lee, Jeonghun Lee, Bradley J. Nelson, Li Zhang, and Hongsoo Choi

Evaluation of the magnetic actuation force on a cilium

The sampling number was introduced to evaluate the behavior of a cilium over one period. The absolute values of the sampling number have no physical meaning and they are considered as modeled time for the magnetic manipulation system. The results calculated are shown in Fig. S1(a-c). These were evaluated by Matlab with a total of 10,000 sampling numbers: 0 to 9,000 for the power stroke and 9,000 to 10,000 for the recovery stroke. The applied field direction (γ), actual cilium angle (θ), and magnetic actuation force (F_m) are shown as a function of the sampling number for one beating cycle in Fig. S1(a-c).

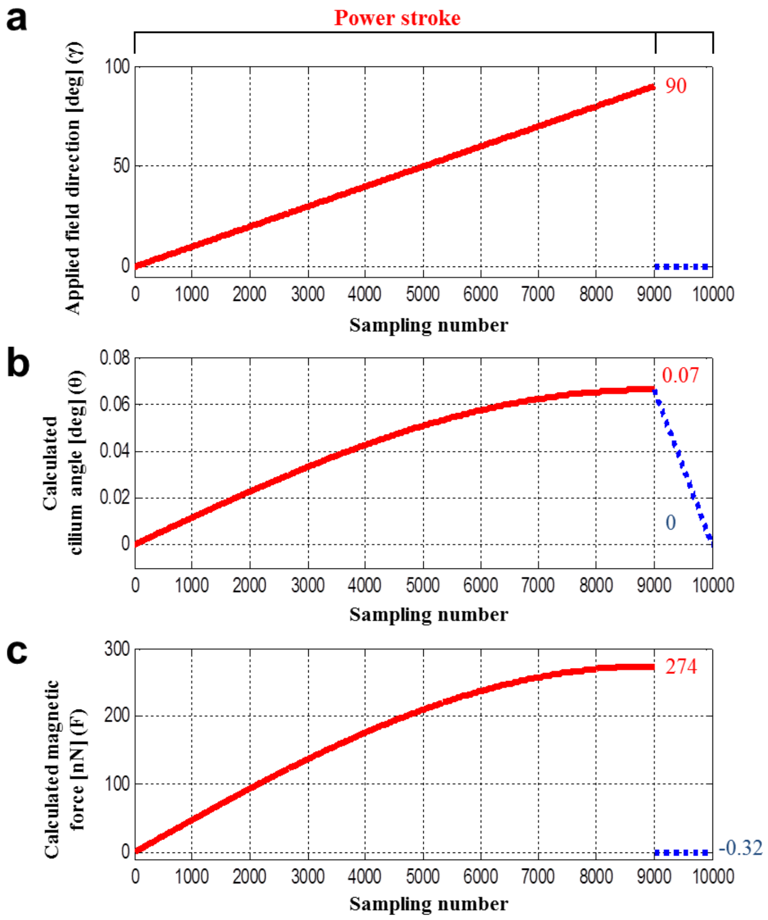

Figure S1. Calculated magnetically actuated force. (a) The modeled applied magnetic field direction (γ), (b) actual cilium angle (θ), and (c) the calculated magnetic actuation force (F_m) (the red line is the power stroke and the blue line is the recovery stroke).

Video S1. Position and orientation control for a ciliary microrobot.
Video S2. Manipulation of a microrobot which drives along each letter of “DGIST”.
Video S3. Targeted particle transportation by the ciliary microrobot.
